# Glanders and Mallein

**Published:** 1895-10

**Authors:** H. D. Gill


### SELECTIONS.

### GLANDERS AND MALLEIN.

BY H. D. GILL, V.S.

SCHUTZ (*Arch. f. Wissensch. u. prakt. Tierheilk.*, xx. 6, 425) investigated lungs from 120 horses suspected of glanders; of these, 92 proved to have the disease in these organs, and, after careful study, he came to the conclusion that glanders of the lungs is never primary. The lungs, however, are the organs first affected in cases of glanders of the external skin or of the mucous membrane.

Since the specific ulcers often heal in the affected places without leaving any easily recognizable scars, and since the inflammatory swelling of the lymph-glands also often subsides, the incorrect assumption of primary affection of the lungs is, in such a case, easy. The anatomical points of differential diagnosis are stated in the original.

Hutyrá and Preisz (*Deut. Ztschr. f. Tiermed. u. vergl. Path.*, xx. 5, 369) have attempted to answer the questions, How great is the diagnostic value of mallein in the recognition of glanders? and, What place does mallein take among the means of diagnosis now known? Sufficient trials have been made to allow a conclusion as to the practical value of the material to be drawn. The authors direct attention to the fact that mallein is not furnished pure, but contained in a mixture of various substances, and the amount of pure mallein in the empirical mixture known as mallein varies. It follows, therefore, that every empirical mallein must be tested, as to its strength, before being used.

According to the cases reported in German literature, mallein has furnished the correct diagnosis in 95.7 per cent. of all cases in which it has been tried. It is to be noted that negative reactions have the same value in the estimate of the material as positive, for autopsy of a considerable proportion of the horses which did not react showed in no case glanders. Study of the records shows that the fever produced by the injection of mallein is typical in infected animals; beginning eight hours after the injection, it attains its maximum in thirteen to fourteen hours, and descends to normal in about twenty-four hours. The fever has no relation to the extent or activity of the disease.

In the cases reported in French literature glanders was found in 99.3 per cent. of those cases in which a positive reaction followed injection. The authors attribute the lack of success experienced by certain opponents of mallein to the poor quality of the material used.

According to Nocard and Schindelka, all horses which undergo a typical reaction in which there is an increase of temperature of 2° C., or more, are to be considered glandered; less reaction does not allow a definite conclusion to be drawn. The authors experimented in a number of cases, and concluded that a reaction of more than 1.5° C., when typical, always indicates glanders. They point out that the temperature curve often has two maxima. The rise is almost always steeper than the descent. The diagnostic value of local and constitutional symptoms is far less than that of the thermic reaction.

According to the view of the authors, there is practically not much to be expected of mallein as a means of treatment.

Other diseases than glanders do not give any reaction with mallein. Finally, the authors give in detail the method used by them in preparing mallein.

Oemler (*Berl. tierärztl. Wochschr.*, 1893) gives the result of eleven series of injections of mallein. The injections, which were given in the usual doses, were repeated two or three times. They were carried out on forty-three horses, which were all more or less suspected of glanders. Only six reacted, and these were shown at autopsy to be affected by the disease. One of the horses which did not react was killed; nothing abnormal was found. The circumstance that in individual cases a reaction has followed the injection of mallein in horses not found to have glanders is, according to the author, not to be urged against the general employment of mallein, for in the usual conditions of practice it is at least very difficult, if not impossible, to confirm surely the absence of glanders.

Schütz (*Arch. f. Wiss. u. prakt. Tierheilk*, xx. 6, 448) had an opportunity to be present at autopsies on fifty-two horses which had been injected with mallein on account of the suspicion of glanders. In fifteen there had been a so-called typical reaction, that is, an increase of the body temperature, about 1.5° C. or over, and a gradual fall thereafter. In seven there was an increase of temperature of 1° to 1.4° C., and in thirty animals there was either none or only a very slight increase of the temperature.

The autopsy showed that no one of the fifty-two horses had suffered from glanders. The nodules, which were often found in the lungs and in the liver of the dead animals, were not considered by the author signs of glanders, on account of their soft structure, and because they were apparently of the same age, as well as because, in spite of the closest investigation, no primary foci could be demonstrated in the mucous membrane of the respiratory passages or in the external skin. The author comes to the conclusion that in this case mallein did not show the asserted specific action.

On another farm mallein injections were made on six suspected horses. In five animals, varying in age from four to nine years, the doses were 0.5 c.cm. each; in one one-year old the dose was 0.3 c.cm. In four of the horses the temperature arose 2° to 3°, and in the fifth 1.5° above the point noted before the injection. The colt did not react. The four horses were killed, and here, too, no signs of glanders were found.

From these trials the author concludes that a typical reaction does not demonstrate that a horse is affected with glanders.

Semmer (*Archiv. des. Scien. Biolog pubal. par. l'Inst. imp. de méd. expér à At. Petersbourg*, 5, 745), in order to decide the question how far mallein could be replaced by other preparations and extracts, tried other bacterial experiments on both healthy and glandered horses with turpentine, tuberculin, extract of the bacillus, prodigiosus, and of the bacterium coli commune and blood-serum of horses affected by glanders. From the experiments the author reports that only the extracts of bacillus prodigiosus and bacillus coli commune gave a reaction like that of mallein, and that these gave a much less intense reaction.

In a horse immunized against glanders the injection of mallein caused only a slight rise in temperature, like that produced by the injection into a healthy animal. An abscess was then produced in the immunized horse by the injection of virulent glanders bacilli, and a mallein injection gave the same result as with the animals affected by glanders, thus demonstrating the presence of the glanders bacilli in the organism of the horse.

The author, associated with Wladimirow, gives also an extremely comprehensive detailed statement of the observations hitherto published as to the action of mallein, and adds many experiments made by himself. He concludes that glanders is demonstrated, if in a horse suspected of glanders and not affected by another disease there is, after the injection of mallein, an elevation of temperature of from 1.5°–3° C., or more, with the formation of a considerable swelling and some constitutional disturbance. After a mallein injection the author observes that there is a lowering of the temperature of about a tenth of a degree, both with glandered and with healthy horses. After some hours the temperature rises, and it attains its maximum eight to fifteen hours after the injection.

In glandered horses the fever reaches usually 2° to 3°, but with healthy horses often 0.7° to 0.8 °, rarely 1° C. In horses which are suffering from some other disease the temperature rises 1° to 2° C., though in them no tumor is observed. The tumor usually begins to appear some hours after the injection and increases in glandered horses for two to three days. In healthy horses it vanishes, if such a tumor is formed at all, on the day after the injection.

